# Notes and News

**Published:** 1891-07-25

**Authors:** 


### Notes and News.

Collected and Communicated.

The Middlesex Hospital, by providing an isolation-room in the out-patients' department for the temporary reception of infectious cases preceding their removal to the fever hospital, has set an example which it would be well if all hospitals were to follow. The careful inspection to which the drainage of the hospital is subjected is also to be commended.

The North-Eastern Hospital for Children is still need of increased support. The twenty-third annua report reveals a deficiency of £1,400 on the accounts, and the amount realised through legacies and donations falls short of that received in 1889 by £2,000. There is no form of charity that appeals more universally to public feeling than a children's hospital, and we hope that a greater number of sympathising friends will be found willing to aid this deserving charity. The closing of the hospital for some weeks in order to reconstruct the drainage will add greatly to the expenditure for this year.

We have received a letter from Mr. A. W. Webster, who misunderstands what we said in criticism of the figures he has prepared, which he claims represent the sums actually received by the London hospitals from the working classes. We do not question the accuracy of the figures as figures. What we said, and what we repeat, is that it is ridiculous to argue that all the money shown in the hospitals' accounts as derived from patients' payments and boxes can really be held to have been given by the working classes alone. As a matter of fact this is far from being the case, as Mr. Webster must, or ought to know. For these reasons we do not think his figures are convincing, or that they prove the point he tried to make.

There is no better administered hospital in the metropolis than the National Hospital for the Paralysed and Epileptic in Queen Square, Bloomsbury. This fact was made apparent to all who attended the opening of the new surgical wing by the Duke and Duchess of Westminster on Saturday last. An inspection of this hospital impresses a visitor with the feeling that if one must be ill then those who find a bed within its walls under such circumstances are, indeed, to be congratulated upon their happy lot. We may congratulate Mr. Burford Rawlings upon the results of his most self-denying and devoted management of this great charity, and can only hope that the £500 required to pay off the debt on the new building will be at once forthcoming. No one who has money to dispense in charity could do better than send a cheque to Mr. Rawlings, at the National Hospital for Paralysed and Epileptic, Queen Square, though we should advise them to visit the institution first, because in that case the contribution is likely to be very much larger than it might otherwise be.

The amateur hypnotist is already making himself objectionable. The punishment meted out for the use of hypnotism to the detriment of a fellow creature took the form of a fine of rather more than £22, in the case of a chemical engineer, named Sandman, of Münsterberg in Germany. He is stated to have hypnotised a girl three times daily, until she eventually became mad, and is now the inmate of a madhouse.

The proposal to erect a Cottage Hospital for medical cases at Walsall has brought to our notice the surprising fact that the town rendered celebrated as the field of Sister Dora's labours amongst the sick, possesses no establishment for the reception of medical cases. This, with a population of 70,000, is a most extraordinary state of affairs. Birmingham, it seems, has supplied the deficiency hitherto, to a large extent. The omission on the part of Walsall is not being rectified too soon. It is proposed to affiliate the new institution with the existing hospital for surgical cases.

Lady Cadogan writes from Chelsea House, Cadogan Place, S.W., that the new convalescent home in connection with the Chelsea Hospital for Women, at St. Leonards-on-Sea, is now open for the reception of patients. The building, which contains 22 beds, stands on its own freehold grounds, in a most healthy and pleasant situation on the West Hill. The charity has no reserve funds, and annual subscriptions are earnestly asked for. Subscribers of £2 2s. per annum and upwards are entitled to a free letter for a three weeks' stay at the home. Full particulars can be obtained from the Secretary, Chelsea Hosp for Women, Fulham Road, S.W.

The enquiry into the allegations made by Robert Washington, the late porter of the Birkenhead Fever Hospital has resulted in a report to the effect that the Sub-committee has carefully enquired into the truth of the charges made, and found that they were for the most part trivial. The only charge of importance was that two male patients in a critical condition were left for one night after half-past eleven p.m. in the sole care of the porter, during which time they both died. This statement on investigation seemed to be in the main true, but it appeared that the Lady Superintendent, though she made every effort to do so, was unable to obtain the services of an additional nurse. Considering the difficulties the Lady Superintendent had to contend with, the Sub-committee found that she did the best she could under the circumstances. The Special Sub-committee, however, was of opinion that she committed an error of judgment in leaving the hospital on the evening of the 10th June for five hours, during a time when she was short-handed and patients were in the hospital, two of them being at that time in a very critical state. The Committee fully exonerated the Matron from the other charges, which were of a most trivial nature.

The annual report of the Middlesex Hospital shows an increase in the number of in-patients in 1890, whilst the out-patients numbered practically the same as during the previous year. The income for the year amounted to £20,634, an increase of £858 on that of 1889, and the expenditure a reduction of £1,473. This decreased expenditure arises principally from minor economies, and reflects great credit on the management of the institution. The capital fund has been augmented by £83,433 during the year. The larger portion of this amount was derived from the munificent bequest of the late Miss Mary Eason. The Samaritan Fund has not been so fortunate. Voluntary contributions to this fund have fallen off considerably, a fact to be much regretted, as this branch of the charity does excellent work. The authorities of the medical school have arranged to make over three per cent. of their gross receipts in return for the amounts expended on rates, taxes, and other items by the hospital on their behalf. The resident staff has been augmented by two salaried officers, a casualty medical, and a casualty surgical officer, for the out-patients department. Miss Newberry, housekeeper to the hospital, has retired, and Miss Lane has been appointed to succeed her.

